# The Neurodynamics of Epilepsy: Synaptic regulation and reversal potential modulation during seizures in a neural field model with conductance-based synapses

**DOI:** 10.1186/1471-2202-14-S1-P47

**Published:** 2013-07-08

**Authors:** Andre DH Peterson, Iven MY Mareels, Hamish Meffin, David B Grayden, Mark J Cook, Anthony N Burkitt

**Affiliations:** 1 NeuroEngineering Lab, Dept. of Electrical & Electronic Engineering, University of Melbourne, Australia; 2 Centre for Neural Engineering, University of Melbourne, Australia; 3 Centre for Clinical Neurosciences, St. Vincent's Hospital, Melbourne, Australia; 4 NICTA Victoria Research Lab, Melbourne, Australia; 5 Bionics Institute, East Melbourne, Australia

## 

Recent experimental results have shown that during the initiation and termination of epileptic seizures there is a significant change in the micro-ionic environment of cortical neurons [1] that affects network balance between excitation and inhibition. This is evident through non-synaptic changes in the micro-ionic environment such as the excitatory and inhibitory reversal potentials and conductances that have been measured over the course of a seizure in an *in vitro *mouse model [2]. Although this phenomenon of a change in reversal potentials during seizures has recently been simulated numerically using a relatively small number of detailed multi-compartmental spiking models [3], it has not yet been modelled using larger scale mesoscopic neural field models within an analytical framework. This is because the majority of these models use current-based synapses, which do not take the reversal potentials into account [4] as they are more difficult to incorporate in mesoscopic models of brain dynamics.

We present an analysis of a neural field model with conductance-based synapses that takes into account the reversal potentials and the nonlinear multiplicative effect that they have on the associated conductance. The conductance-based synapses model is derived, analysed and juxtaposed with the current-based synapses model and the results interpreted physiologically.

A comparative bifurcation analysis of both models reveals that there are significant differences in the oscillatory behaviours that correspond to epileptic seizures [4]. In the conductance-based synapses model, there are endogenous anti-epileptic regulatory or control mechanisms that operate on the synaptic scale, whereas previously these were thought to be mainly on the network level; for example, in terms of feedback, feed-forward and surround inhibition [5]. Further, upon modulation of the reversal potentials, the new model exhibits seizure behaviour that initiates and terminates due to non-synaptic ionic mechanisms, similar to that measured in recent experiments [1].

Seizure dynamics in the brain are modulated by both synaptic regulatory mechanisms and non-synaptic homeostatic mechanisms, which play key roles during seizure initiation, spread and termination [5]. These mechanisms have been investigated using a novel analytical neural field model which has provided insights into understanding epileptic brain dynamics that are not currently observable in electrophysiological experiments and numerical simulations alone.
